# The diagnostic performance of deep-learning-based CT severity score to identify COVID-19 pneumonia

**DOI:** 10.1259/bjr.20210759

**Published:** 2021-11-30

**Authors:** Anna Sára Kardos, Judit Simon, Chiara Nardocci, István Viktor Szabó, Norbert Nagy, Renad Heyam Abdelrahman, Emese Zsarnóczay, Bence Fejér, Balázs Futácsi, Veronika Müller, Béla Merkely, Pál Maurovich-Horvat

**Affiliations:** 1 Medical Imaging Centre, Semmelweis University, Budapest, Hungary; 2 MTA-SE Cardiovascular Imaging Research Group, Heart and Vascular Center, Semmelweis University, Budapest, Hungary; 3 Department of Pulmonology, Semmelweis University, Budapest, Hungary

## Abstract

**Objective:**

To determine the diagnostic accuracy of a deep-learning (DL)-based algorithm using chest computed tomography (CT) scans for the rapid diagnosis of coronavirus disease 2019 (COVID-19), as compared to the reference standard reverse-transcription polymerase chain reaction (RT-PCR) test.

**Methods:**

In this retrospective analysis, data of COVID-19 suspected patients who underwent RT-PCR and chest CT examination for the diagnosis of COVID-19 were assessed. By quantifying the affected area of the lung parenchyma, severity score was evaluated for each lobe of the lung with the DL-based algorithm. The diagnosis was based on the total lung severity score ranging from 0 to 25. The data were randomly split into a 40% training set and a 60% test set. Optimal cut-off value was determined using Youden-index method on the training cohort.

**Results:**

A total of 1259 patients were enrolled in this study. The prevalence of RT-PCR positivity in the overall investigated period was 51.5%. As compared to RT-PCR, sensitivity, specificity, positive predictive value, negative predictive value and accuracy on the test cohort were 39.0%, 80.2%, 68.0%, 55.0% and 58.9%, respectively. Regarding the whole data set, when adding those with positive RT-PCR test at any time during hospital stay or “COVID-19 without virus detection”, as final diagnosis to the true positive cases, specificity increased from 80.3% to 88.1% and the positive predictive value increased from 68.4% to 81.7%.

**Conclusion:**

DL-based CT severity score was found to have a good specificity and positive predictive value, as compared to RT-PCR. This standardized scoring system can aid rapid diagnosis and clinical decision making.

**Advances in knowledge:**

DL-based CT severity score can detect COVID-19-related lung alterations even at early stages, when RT-PCR is not yet positive.

## Introduction

The coronavirus disease 2019 (COVID-19) caused by the severe acute respiratory syndrome– associated coronavirus 2 (SARS-CoV-2) was declared as a pandemic by the World Health Organization (WHO).^1^ In Hungary, the first COVID-19 cases were discovered in the beginning of March 2020.^2^ The virus is harboured most commonly with little or no symptoms but can also lead to a rapidly progressive and often fatal pneumonia.^3–5^ With infection numbers over 100 million, the disease is a dangerous public health threat.^6,7^

The rapid rate of spread has strained healthcare systems worldwide due to shortages in key protective equipment and point-of-care testing methodologies, including reverse-transcription polymerase chain reaction (RT-PCR). Even if RT-PCR testing becomes more available, challenges remain, such as limited sensitivity in the early stages of the disease, long processing time placing a strain on the holding units where patients are kept before being sent to a normal or COVID-19 ward and variabilities in test techniques and qualities.^8–10^ The high number of false-negative cases might lead to a delay in therapy and an increase in in-hospital spread.^11^

Chest computed tomography (CT) can detect certain characteristic COVID-19 manifestations in the lung, such as peripheral ground glass opacities, interstitial changes and multifocal patchy consolidations.^12–14^ However, there is controversy among recommendations for the use of CT as a surrogate diagnostic test for COVID-19.^15^ COVID-19 Reporting and Data System (CO-RADS) has been developed in order to ensure a standardized CT reporting score.^16^ CO-RADS assigns a score of 0–5 based on the CT findings and a score of 6 in RT-PCR confirmed COVID-19 cases. However, regarding the reproducibility of this scoring system, significant differences could be between physicians. Moreover, the CO-RADS score 6 given to those with known RT-PCR positivity does not provide information on the extent of the affected lung area.^17^ The gap between the number of CT examinations and the available radiologists is increasing constantly, creating a demand for techniques that can aid radiologists. Artificial intelligence (AI) using deep-learning (DL) algorithms has the potential to improve diagnostic accuracy, lowering false-negative rate and aid the rapid evaluation of chest CT scans.^18–22^ The aim of this study was to determine the diagnostic accuracy of a DL-based algorithm in the detection of COVID-19.

## Methods

The study followed the Standards for Reporting of Diagnostic Accuracy Studies (STARD) criteria.^23^

### Patient selection and data collection

This retrospective analysis included a consecutive series of patients with suspected COVID-19 who underwent RT-PCR test and chest CT examination for the diagnosis of COVID-19 in any Department of the Semmelweis University, Budapest, Hungary between April and December 2020. Patients with non-diagnostic image quality for DL-based CT evaluation were excluded from the analysis.

### CT acquisition protocol and image reconstruction

Chest CT scans were obtained using a 128-slice CT scanner (Philips Incisive, Philips Healthcare, Cleveland, Ohio) in the supine position during inspiratory breath hold. The CT acquisition protocol included a peak tube voltage of 120 kV, automatic tube current modulation (300–500 mAs), slice thickness of 1 mm and reconstruction increment 0.85 with a collimation of 64 × 0.625. Infection control and prevention were considered in all cases. Images were reconstructed using standard lung filters.

### CT image analysis

**Figure 1.**
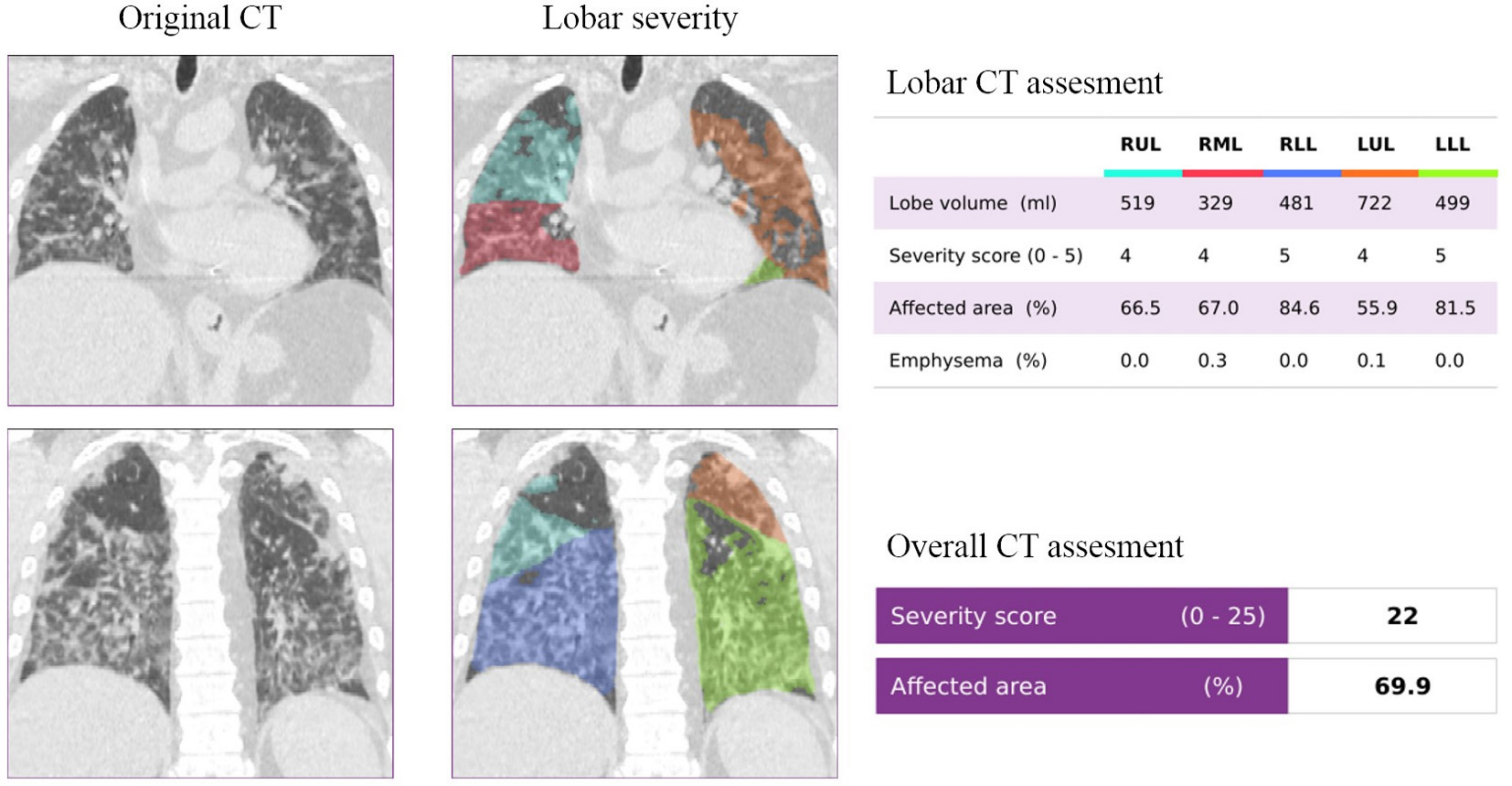
Representative example of a COVID-19 patient with a severity score of 22. The original and DL-assessed chest CT of a 76-year-old male patient, who had a positive RT-PCR test at the time of the CT examination.

**Figure 2.**
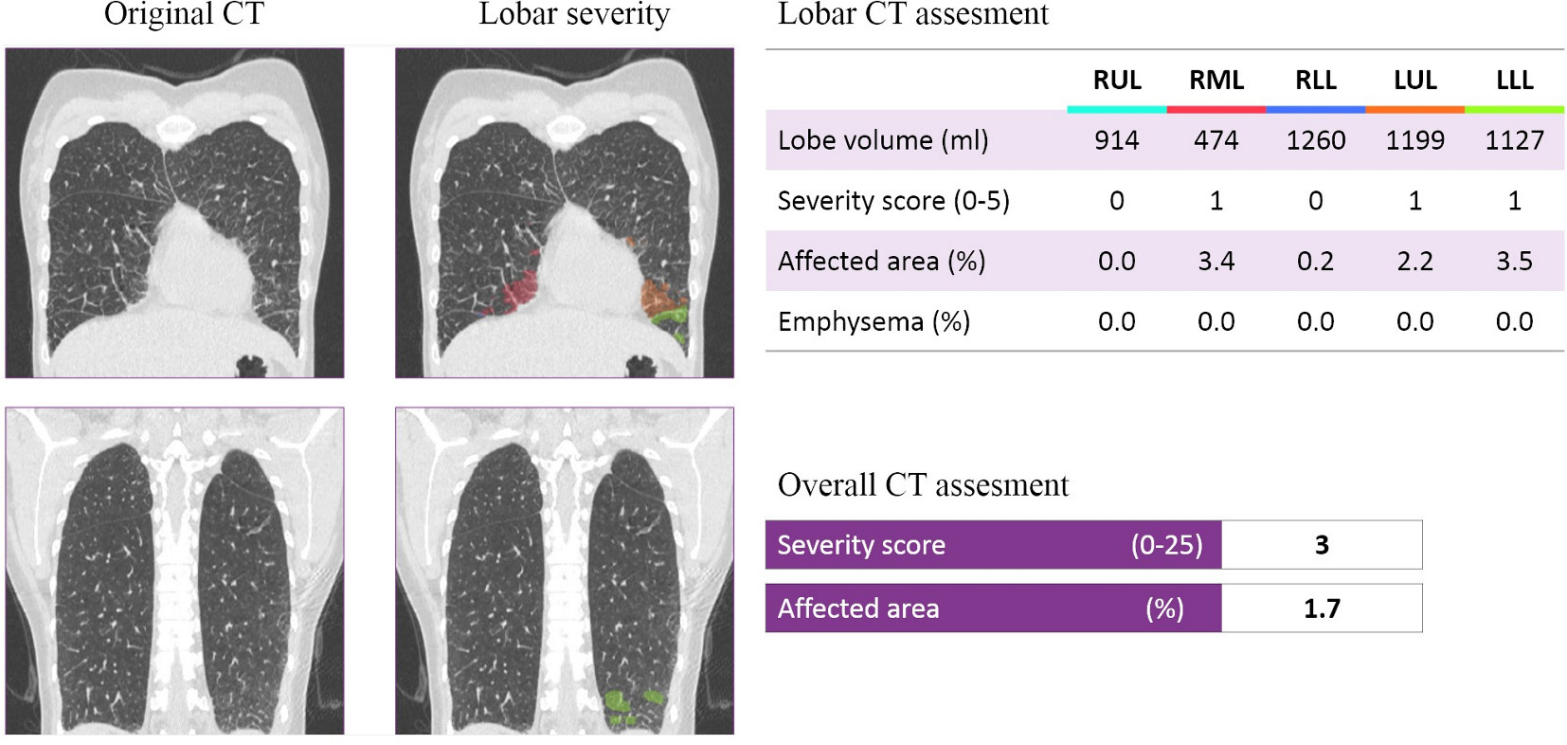
Representative example of a COVID-19 patient with a severity score of 3. The original and DL-assessed chest CT of a 41-year-old female patient, who tested negative for COVID-19.

CT quantification of pulmonary parenchyma was performed using the CAD4COVID-CT software (Thirona, Nijmegen, the Netherlands). CAD4COVID-CT is an AI-based software package that is offered free of charge during the COVID-19 pandemic to assist healthcare professionals in their daily tasks. The software automatically quantifies the lobar extent of COVID-19 severity from inspiratory CT scans using state-of-the-art deep-learning techniques. The quantitative output of the software includes the volume (ml) and the COVID-19 affected area (%) for each lobe, and expresses this information as a lobar COVID-19 severity score between 0 and 5. The total severity score is expressed as the sum of each lobar score and therefore ranges between 0 and 25. Considering that the software only uses the CT images, clinical information and reference standard results were unavailable. CAD4COVID-CT is CE 0344 certified as a Class IIa medical device and is permitted to be used in the USA by the Food and Drug Administration (FDA). Examples can be seen in Figures 1 and 2.

### Reference standard

For RT-PCR test, pharyngeal and nasal samples were used. Patients with positive RT-PCR result were considered to be infected with COVID-19, whereas patients with negative RT-PCR result were considered not infected. Those with initial negative RT-PCR and symptoms indicating COVID-19 were retested within 48 h. Clinical information and index test results were not taken into consideration by RT-PCR testing.

### Statistical analysis

The data set was randomly split into two cohorts: a training set (40%) and a test set (60%). Using the training cohort, an optimal cut-off value for DL-based CT severity score was obtained based on Youden-index method. Using RT-PCR results as the gold standard, sensitivity, specificity, positive predictive value, negative predictive value and accuracy of DL-based CT severity score were determined for both cohorts.

An additional analysis was also performed on the whole data set: for patients with positive RT-PCR but negative CT result, CO-RADS categories and clinical symptoms were collected in order to determine the clinical manifestation of the disease. For those patients with positive CT result but negative RT-PCR, subsequent RT-PCR results and/or final diagnosis of the patients were collected. Based on these findings, the patients were reclassified into real COVID-19 cases (those with positive RT-PCR test at any time during hospital stay or “COVID-19 without virus detection”, as the final diagnosis) and non-COVID-19 cases. Statistical analysis was performed in R environment (v. 4.0.3).

## Results

### Diagnostic accuracy of DL-based CT severity score as compared with RT-PCR

**Figure 3.**
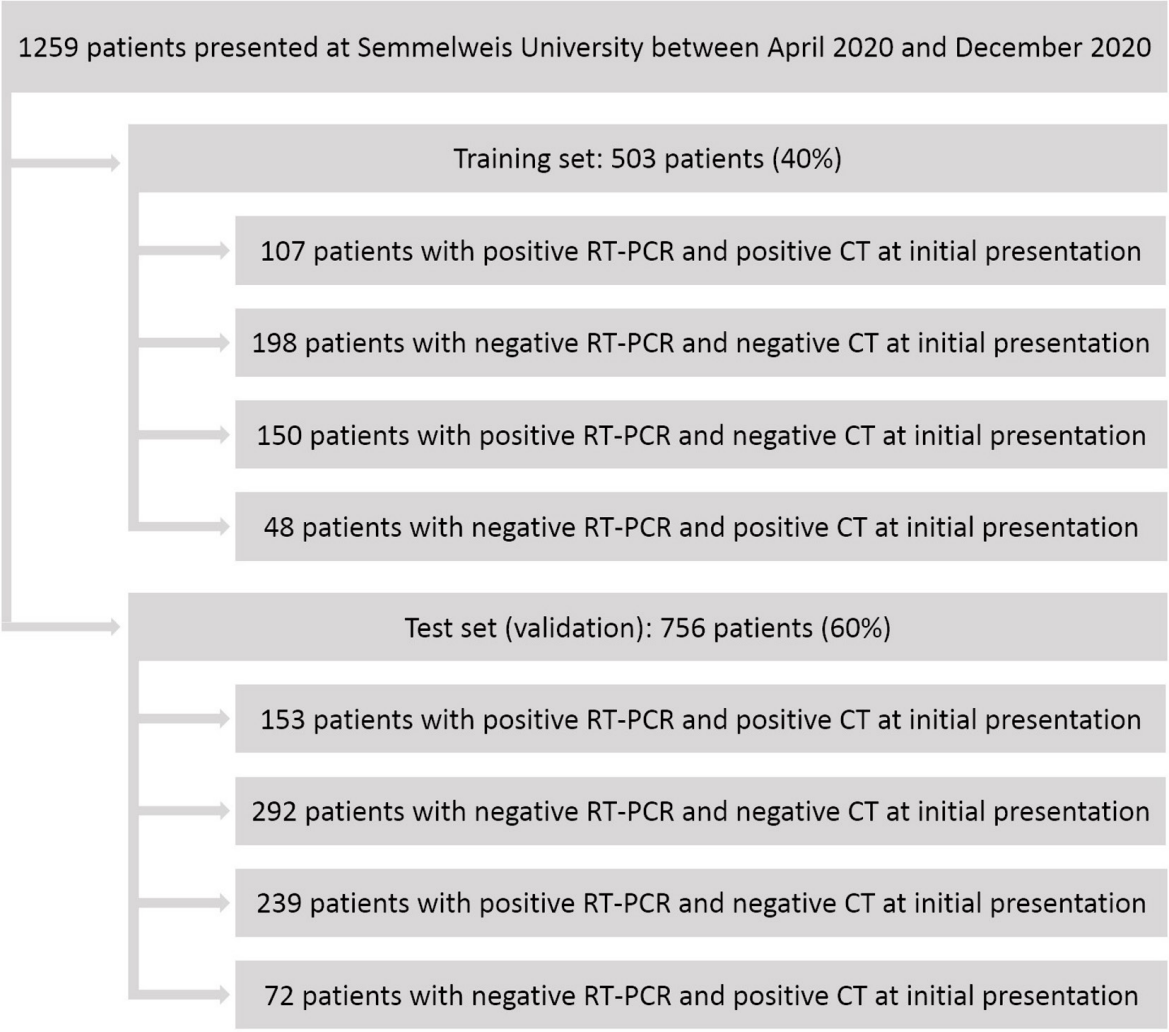
Flowchart of this study. RT-PCR, reverse-transcription polymerase chain reaction.

A total of 1259 patients were enrolled in this study. The training cohort consisted of 503 patients (40% of the data), while the test cohort consisted of 756 patients (60%). The prevalence of RT-PCR positivity in the training and the test set was 51.1% and 51.9%, respectively (*p* = 0.837), as detailed in Supplementary Table 1. Optimal cut-off value for the DL-based CT severity score was 9. At this cut-off value, 107 cases were true positive, 198 cases were true negative in the training cohort, while in the test cohort, 153 cases were true positive and 292 cases were true negative, as compared to RT-PCR, as reported in Figure 3. Sensitivity, specificity, positive predictive value, negative predictive value and accuracy for the training cohort were 41.6%, 80.5%, 69.0%, 56.9% and 60.6%, respectively. Whereas for the test cohort sensitivity, specificity, positive predictive value, negative predictive value and accuracy were 39.0%, 80.2%, 68.0%, 55.0% and 58.9%, respectively. Detailed results on the diagnostic performance of DL-based CT severity score can be seen in Table 1.

**Table 1.** Diagnostic performance of DL-based CT severity score

	Training cohort (*n* = 503)	Test cohort (*n* = 756)
Prevalence, %	51.1	51.9
Sensitivity, % (95% CI)	41.6% (35.5–47.9%)	39.0 (34.2–44.1)
Specificity, % (95% CI)	80.5 (75.0–85.2)	80.2 (75.8–84.2)
Positive predictive value, % (95% CI)	69.0 (61.1–76.2)	68.0 (61.5–74.0)
Negative predictive value, % (95% CI)	56.9 (51.5–62.2)	55.0 (50.9–59.3)
Accuracy, % (95% CI)	60.6 (56.2–64.9)	58.9 (55.3–62.4)

The prevalence of COVID-19 and the diagnostic performance of DL-based chest CT severity score were determined on the whole data set separately in the first (between April and June 2020) and second (between August and December 2020) waves of the disease. During the spring wave, the prevalence of COVID-19 was 25.1%, whereas during the fall wave, it was 55.8%. Due to the lower prevalence during the first wave, this period was associated with better specificity and negative predictive value compared to the second wave, as reported in Table 2.

**Table 2.** Diagnostic performance of DL-based CT severity score

	Total investigated time (April–December 2020)	Spring wave (April–June 2020)	Autumn wave (August–December 2020)
Prevalence, %	51.5	25.1	55.8
Sensitivity, % (95% CI)	40.1 (36.3–43.9)	18.2 (8.2–32.7)	41.7 (37.7–45.7)
Specificity, % (95% CI)	80.3 (76.9–83.4)	84.7 (77.4–90.4)	79.1 (75.2–82.7)
Positive predictive value, % (95% CI)	68.4 (63.5–73.1)	28.6 (13.2–48.7)	71.6 (66.6–76.2)
Negative predictive value, % (95% CI)	55.7 (52.4–59.6)	75.5 (67.7–82.2)	51.8 (48.1–55.5)
Accuracy, % (95% CI)	59.6 (56.8–62.3)	68.0 (60.5–74.8)	58.2 (55.2–61.2)

### Subanalysis of false-negative and false-positive cases

The subanalysis was performed on the whole data set. The low sensitivity and negative predictive value can be explained by the fact that in many cases, despite RT-PCR positivity, COVID-19 has no or minimal lung manifestations. When examining the CO-RADS classification, these patients mainly had a score of 1, 2 or 6. Regarding the complaints of the false-negative patients, only 32.1% had shortness of breath. Examining the CO-RADS classification of false-positive patients, they mainly had a score of 4 or 5; however, the RT-PCR test performed at the time of the CT examination was negative and 66.4% had shortness of breath. Detailed results of the presenting symptoms can be seen in Table 3.

**Table 3.** . Symptoms of COVID-19 suspected patients at the time of hospital admission. False-negative cases are patients with positive RT-PCR and negative CT results. False-positive cases are patients with negative RT-PCR and positive CT results

Symptom	False-negative cases *n* = 389	False-positive cases *n* = 120
Fever and chills, %	49.1	40.2
Shortness of breath, %	32.1	66.4
Dry cough, %	41.8	54.1
Productive cough, %	11.9	16.6
Loss of taste, %	10.1	2.8
Loss of smell, %	9.3	2.3
Muscle or joint pain, %	13.9	4.1
Headache, %	5.5	2.2
Nausea, vomiting, %	11.4	4.3
Diarrhea, %	8.0	4.7

**Figure 4.**
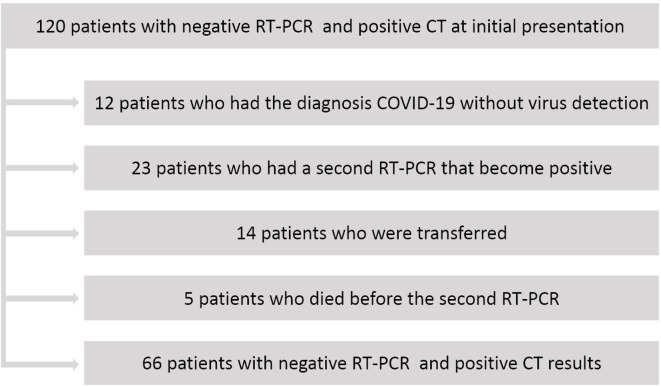
Subanalysis of patients with negative RT-PCR and positive CT at initial presentation (false-positive patients).

When analyzing the data of the 120 false-positive cases, “COVID-19 disease without virus detection” was the final diagnosis in 12 patients. They were placed to COVID-19 care unit. Despite exhibiting a typical chest CT for COVID-19, 37 patients were transferred to a non-COVID-19 care unit due to RT-PCR negativity. In 23 patients, the second RT-PCR was positive and 14 patients were lost to follow-up, with no access to their subsequent medical reports. Another five patients died before the second RT-PCR test, as reported in Figure 4.

Accordingly, the diagnostic performance of the DL-based chest CT severity score was re-examined for the detection of COVID-19 disease. To this end, the cases where collection of post-hospital admission data (*n* = 19) was not possible and were excluded from the analysis. Patients whose final diagnosis was “COVID-19 without virus detection” (*n* = 12) or only the second RT-PCR was positive (*n* = 23) were added to the true-positive cases. Therefore, in the new analysis, the number of false-positive cases was reduced from 120 to 66. Accordingly, the specificity of DL-based CT severity score increased from 80.3% to 88.1% and the positive predictive value increased from 68.4% to 81.7%. Altogether, the diagnostic accuracy of RT-PCR improved from 97.4% (95%CI: 96.1–98.0%) to 99.7% (95%CI: 99.6–99.8%;) when DL-based CT severity score was added (*p* < 0.001).

## Discussion

The results suggest that DL-based CT severity score has a good specificity and positive predictive value on both training and test cohort, compared to RT-PCR with proven ability to detect COVID-19-related lung alterations even at early stages, when RT-PCR is negative. Moreover, when combined with DL-based CT severity score, the diagnostic accuracy of RT-PCR improved from 97.4% to 99.7% to identify COVID-19 using the clinical diagnosis as the reference standard.

During the COVID-19 pandemic, chest CT proved to be a useful tool for the diagnosis and follow-up of patients with COVID-19. CT has a faster turnaround time than RT-PCR test and it can provide more detailed information on the extent of lung involvement, which reflects disease severity and prognosis.^24^ Moreover, we can perform a CT pulmonary angiogram to rule out pulmonary vascular thrombosis, which could trigger worsening clinical condition.^25^ At the same time, an increased number of COVID-19 cases have placed an excessive burden on radiologists. A CT-based AI system may have the potential to assist in the early diagnosis, monitoring and treatment planning.^26,27^ According to Ji et al, there is a significant positive correlation between COVID-19 mortality and healthcare burden.^28^

Since the beginning of the COVID-19 pandemic several studies aimed to establish DL-based methods for the diagnosis of COVID-19 on CT scans and sought to investigate the diagnostic power of these systems, as compared to RT-PCR.^17,19,29,30^ Mei et al developed an AI system derived from heterogeneous multinational training data. The test set included 279 cases.^17^ Wang et al built an AI system that provides the probability of infection to rapidly detect COVID-19 pneumonia. They tested the system on 170 cases.^29^ Wang et al established a DL model for distinguishing COVID-19 and typical pneumonia. The external test set consisted of 290 cases.^19^ Wang et al generated a DL system to help COVID-19 diagnostic and prognostic analysis. The system used a training data set including CT scans of 924 cases with COVID-19 and 342 cases of other pneumonia, and the two test sets included 194 cases with COVID-19 and 193 cases of other pneumonia.^30^ In these studies, specificity of AI-based algorithms ranged from 74% to 83%. In line with this data, the CAD4COVID-CT DL-based algorithm had similar capability to correctly identify COVID-19 negative cases with a specificity of 80.2% in the test cohort. However, while sensitivity values varied between 67% and 83% in these studies, our DL-based algorithm had a sensitivity of 39.0% on the test set. This difference might be due to the less strict inclusion criteria since our centre had the capacity to perform a CT not only for patients with moderate-to-severe clinical features and a high pretest probability of disease but also to those patients with mild or no respiratory symptoms, which resulted in a high number of false-negative CT results.^31^

Moreover, since RT-PCR may yield false-negative results at the early stages of COVID-19 infection, we aimed to determine the diagnostic performance of the DL-based CT severity score in the detection of COVID-19. For this, patients whose final diagnosis was “COVID-19 without virus detection” or those whose second RT-PCR was positive were considered as true-positive cases. “COVID-19 without virus detection” was the diagnosis when the patient had a clinical presentation or chest CT results that made COVID-19 the probable diagnosis, and there was no alternative diagnosis explaining the symptoms or the characteristic lung involvement. In this analysis, specificity increased by 7.8% and positive predictive value by 13.3%. Furthermore, standardized CT scoring systems have been introduced to improve communication between radiologists and other healthcare providers by enabling fast and consistent clinical decision-making, a valuable asset in these enduring times of crisis.

The DL-based chest CT severity score may support radiologists in standardized CT reporting during loaded periods, especially in patients with respiratory symptoms. It can aid the diagnosis of COVID-19, lowering false-negative rates, and provides information not only on the presence of lung involvement but also the extent of it.

There are limitations to this study. First, data from only one medical center were used. Second, this DL-based severity score does not distinguish between the different types of lesions. Another limitation is the bias towards patients with COVID-19. On chest CT images, ground glass opacities and other features are non-specific, which could limit the usefulness of the DL-based CT severity score to differentiate COVID-19 pneumonia from other causes of respiratory failure. Moreover, for the determination for an optimal cut-off value for the DL-based severity score, Youden-index method was used. Notably, the optimal cut-off value changes with the prevalence of the disease.
